# Newborn screening analytes and structural birth defects among 27,000 newborns

**DOI:** 10.1371/journal.pone.0304238

**Published:** 2024-07-05

**Authors:** Philip J. Lupo, Natalie P. Archer, Rachel D. Harris, Lisa K. Marengo, Jeremy M. Schraw, Adrienne T. Hoyt, Susan Tanksley, Rachel Lee, Margaret Drummond-Borg, Debra Freedenberg, Priya B. Shetty, A. J. Agopian, Charles Shumate, Sonja A. Rasmussen, Peter H. Langlois, Mark A. Canfield

**Affiliations:** 1 Department of Pediatrics, Section of Hematology-Oncology, Baylor College of Medicine, Houston, Texas; 2 Texas Children’s Cancer and Hematology Centers, Texas Children’s Hospital, Houston, Texas; 3 Birth Defects Epidemiology and Surveillance Branch, Environmental Epidemiology and Disease Registries Section, Texas Department of State and Health Services, Austin, Texas; 4 Department of Health and Human Performance, University of Houston, Houston, Texas; 5 Laboratory Services Section, Texas Department of State Health Services, Austin, Texas; 6 Department of Epidemiology, Human Genetics and Environmental Sciences, University of Texas Health Science Center at Houston (UTHealth) School of Public Health, Houston, Texas; 7 Johns Hopkins University School of Medicine, Baltimore, Maryland; 8 Department of Epidemiology, Human Genetics and Environmental Sciences, University of Texas Health Science Center at Houston (UTHealth), School of Public Health, Austin, Texas; Canakkale Onsekiz Mart University School of Medicine, TURKEY

## Abstract

**Background:**

Emerging evidence suggests newborn screening analytes may yield insights into the etiologies of birth defects, yet no effort has evaluated associations between a range of newborn screening analytes and birth defects.

**Methods:**

This population-based study pooled statewide data on birth defects, birth certificates, and newborn screening analytes from Texas occurring between January 1, 2007 and December 31, 2009. Associations between a panel of thirty-six newborn screening analytes, collected by the statewide Texas Newborn Screening Program, and the presence of a birth defect, defined as at least one of 39 birth defects diagnoses recorded by the Texas Birth Defects Registry, were assessed using regression analysis.

**Findings:**

Of the 27,643 births identified, 20,205 had at least one of the 39 birth defects of interest (cases) as identified by the Texas Birth Defects Registry, while 7,438 did not have a birth defect (controls). Among 1,404 analyte-birth defect associations evaluated, 377 were significant in replication analysis. Analytes most consistently associated with birth defects included the phenylalanine/tyrosine ratio (N = 29 birth defects), tyrosine (N = 28 birth defects), and thyroxine (N = 25 birth defects). Birth defects most frequently associated with a range of analytes included gastroschisis (N = 29 analytes), several cardiovascular defects (N = 26 analytes), and spina bifida (N = 23 analytes).

**Conclusions:**

Several significant and novel associations were observed between newborn screening analytes and birth defects. While some findings could be consequences of the defects themselves or to the care provided to infants with these defects, these findings could help to elucidate mechanisms underlying the etiology of some birth defects.

## 1. Introduction

Newborn screening (NBS) is an important public health program that tests for the presence of health disorders using a heel stick blood sample collected soon after birth (typically within 24-hours) [1]. NBS programs first began in the 1960s in the United States and have since improved the ability to provide early disease detection, leading ultimately to increased secondary prevention strategies of screened disorders [2]. Each year in the United States, 4 million newborns are screened for hearing loss and some metabolic, endocrine, and genetic disorders such as phenylketonuria, hypothyroidism, cystic fibrosis, and spinal muscular atrophy. Notably, there is emerging evidence that NBS analytes are also associated with conditions not included in traditional screening programs, including autism and some birth defects [3–5].

Each year in the United States, more than 120,000 infants are born with birth defects, which remain the leading cause of death among infants. Furthermore, these children often face multiple corrective surgeries and chronic health conditions, which can limit their quality of life [6–8]. In spite of their clinical importance, more than 80% of birth defects are of unknown etiology, which limits screening and prevention strategies [9]. Complicating the diagnostic journey of these children, some birth defects such as choanal atresia, which is characterized by a narrowing of the nasal cavity that results in breathing difficulty, are not evident at birth but can be life-threatening if not treated. Because of this, leveraging data from NBS programs may provide novel insights into the etiologies of birth defects and help improve early detection of certain birth defects.

Two previous studies in Texas have demonstrated associations between one NBS analyte, thyroxine, and two birth defects, craniosynostosis [4] and choanal atresia [3]. However, there have been no large-scale, population-based efforts to evaluate associations between a comprehensive panel of NBS analytes and a range of birth defects. Therefore, we conducted a phenotypic spectrum analysis to evaluate associations between a range of NBS analytes and birth defects evaluated in the Texas Birth Defects Registry (TBDR).

## 2 Methods

### 2.1 Study population

Data on liveborn infants with birth defects born to Texas residents during the period 2007–2009 were obtained from the TBDR. The TBDR is a population-based, active surveillance system that has monitored births throughout the state of Texas since 1999. All cases had one or more ‘‘definite” birth defect diagnoses documented during their first year of life coded according to the Centers for Disease Control and Prevention modification of the British Paediatric Association Classification of Diseases. We included a total of 39 birth defects of interest (38 structural birth defects and trisomy 21) based on the National Birth Defects Prevention Network list of reported birth defects (S1 Table) [10]. The 39 birth defects of interest were further categorized into the following defect groups: central nervous system (CNS), cardiac, chromosomal, eye or ear, gastrointestinal, genitourinary, musculoskeletal, oral clefts, and respiratory (S2 Table). As NBS analytes may differ based on total parenteral nutrition (TPN) [11, 12], we did not include cases with TPN in this analysis as it might bias our results. For this analysis, controls were unaffected births not present in the TBDR, drawn from Texas birth certificates (obtained from the Texas Department of State Health Services Center for Health Statistics) for the same study period (2007–2009). Infants with blood transfusions after delivery were excluded from the study. Data were accessed on September 7,2021 for research purposes. The study protocol is provided as S1 File.

### 2.2 Data collection

Information on 36 NBS analytes collected from each infant within 7 days of delivery was obtained from the Texas NBS Program (S3 Table). Texas routinely performs two NBS screens on each infant. The first screen usually occurs within 24 to 48 hours but no later than 7 days after delivery. The second screen usually occurs 7 to 14 days after delivery. For this analysis, we focused on the first screen results. As it is possible that infants admitted to the neonatal intensive care unit (NICU) may have samples collected earlier (e.g., prior to 24 hours), as well as multiple samples collected, we utilized the latest screen if multiple screens occurred within the 7 days following birth to minimize differences in collection timing between infants admitted to the NICU compared to those who were not. Finally, NBS analytes were further categorized as: amino acids, fatty acids, hormones, and organic acids.

Data on maternal demographic and infant characteristics were obtained from the Center for Health Statistics, collected from vital records, and included maternal age at delivery (<20, 20–24, 25–29, 30–34, 35–39, ≥40 years); maternal race/ethnicity (Hispanic, non-Hispanic Black, non-Hispanic White, or other); infant sex (male or female); birth weight (grams); and gestational age (weeks).

### 2.3 Statistical analysis

Summary statistics for the mothers and infants were reported by case-control status. Next, we used a two-stage phenome-wide association study (pheWAS) approach [13–15] to evaluate associations between the 36 NBS analytes and the 39 selected birth defects. Specifically, 70% of the cases and controls were randomly sampled into a discovery dataset, while the remaining 30% comprised the replication dataset. The NBS analyte levels were categorized into quartiles, stratified by newborn analyte screening batch, which created batch-dependent quartile values for analysis. To allow testing for trends, the NBS quartiles were also analyzed as continuous variables for all analyses. Logistic regression was used to calculate an odds ratio (OR), 95% confidence interval, and p-value for each analyte-birth defect pair. Based on previous assessments [3, 4], models were adjusted for maternal age, infant sex, infant birthweight (grams), and gestational age at birth (weeks). As we conducted 1,404 comparisons, we adjusted for multiple testing in the discovery analysis using the Bonferroni correction, setting the significance threshold at 3.6 x 10^−5^. In analysis of the remaining 30% replication dataset, results with p-values < 0.05 and effect estimates in the same direction as with the discovery dataset were reported as significant.

#### 2.3.1 Sensitivity analyses

We conducted a sensitivity analysis to evaluate the impact of the number defects on the associations tested. Specifically, we evaluated associations among children with only isolated birth defects (i.e., children without co-occurring major birth defects or, in the case of cardiac defects, children without major extra-cardiac defects). These analyses were intended to address both the potential influence of genetic syndromes and potential heterogeneity among nonsyndromic cases.

All data analyses were conducted using SAS, version 9.4 (Cary, NC) and data visualization was performed in R version 4.2.1. This study was approved by the institutional review boards (IRBs) at the Texas Department of Health Services and Baylor College of Medicine. This study was performed in accordance with the Declaration of Helsinki. Written informed consent was not obtained because this was a secondary analysis of existing data.

## 3 Results

A total of 27,643 births were identified and included in our analyses (N = 20,205 with at least one diagnosed birth defect and N = 7,438 without birth defects). Demographic characteristics of the cohort by case/control status are presented in Table 1. Overall, cases were more likely to be male, with a lower birth weight (<3,100 grams), and younger gestational age (<39 weeks).

**Table 1:** Study population characteristics by case/control status (N = 27,643) (Texas, 2007–2009).

	Birth Defect Status		
	Cases	Controls	Total
	(n = 20,205)	(n = 7,438)	(n = 27,643)
**Infant Sex, n(%)**			
Male	12,672 (62.7)	3,796 (50.1)	16,469 (55.5)
Female	7,533 (37.3)	3,642 (49.0)	11,175 (44.4)
**Maternal Age, n(%)**			
10–19	2,691 (13.3)	1,013 (13.6)	3,704 (13.4)
20–24	5,434 (26.9)	1,999 (26.9)	7,433 (26.9)
25–29	5,209 (25.8)	2,127 (28.6)	7,336 (26.5)
30–34	3,990 (19.7)	1,444 (19.4)	5,434 (19.7)
35–39	2,192 (10.8)	707 (9.5)	2,899 (10.5)
40+	688 (3.4)	148 (2.0)	836 (3.0)
Missing	1 (0.0)	0 (0.0)	1 (0.0)
**Maternal Race/Ethnicity, n(%)**			
Non-Hispanic White	7,398 (36.6)	2,504 (33.7)	9,902 (35.8)
Non-Hispanic Black	2,023 (10.0)	892 (12.0)	2,915 (10.5)
Hispanic	10,079 (49.0)	3,686 (49.6)	13,765 (49.8)
Other	690 (3.4)	349 (4.7)	1,039 (3.8)
Missing	15 (0.1)	7 (0.1)	22 (0.0)
**Birth Weight (*g*)** ^**a**^	3,099.9 (693.8)	3,281.2 (517.7)	3,148.7 (656.0)
**Gestational Age (weeks)** ^**a**^	38.3 (2.9)	39.0 (2.3)	38.5 (2.7)

^a^ Mean(Standard Deviation)

### 3.1 All birth defects

**Fig 1:**
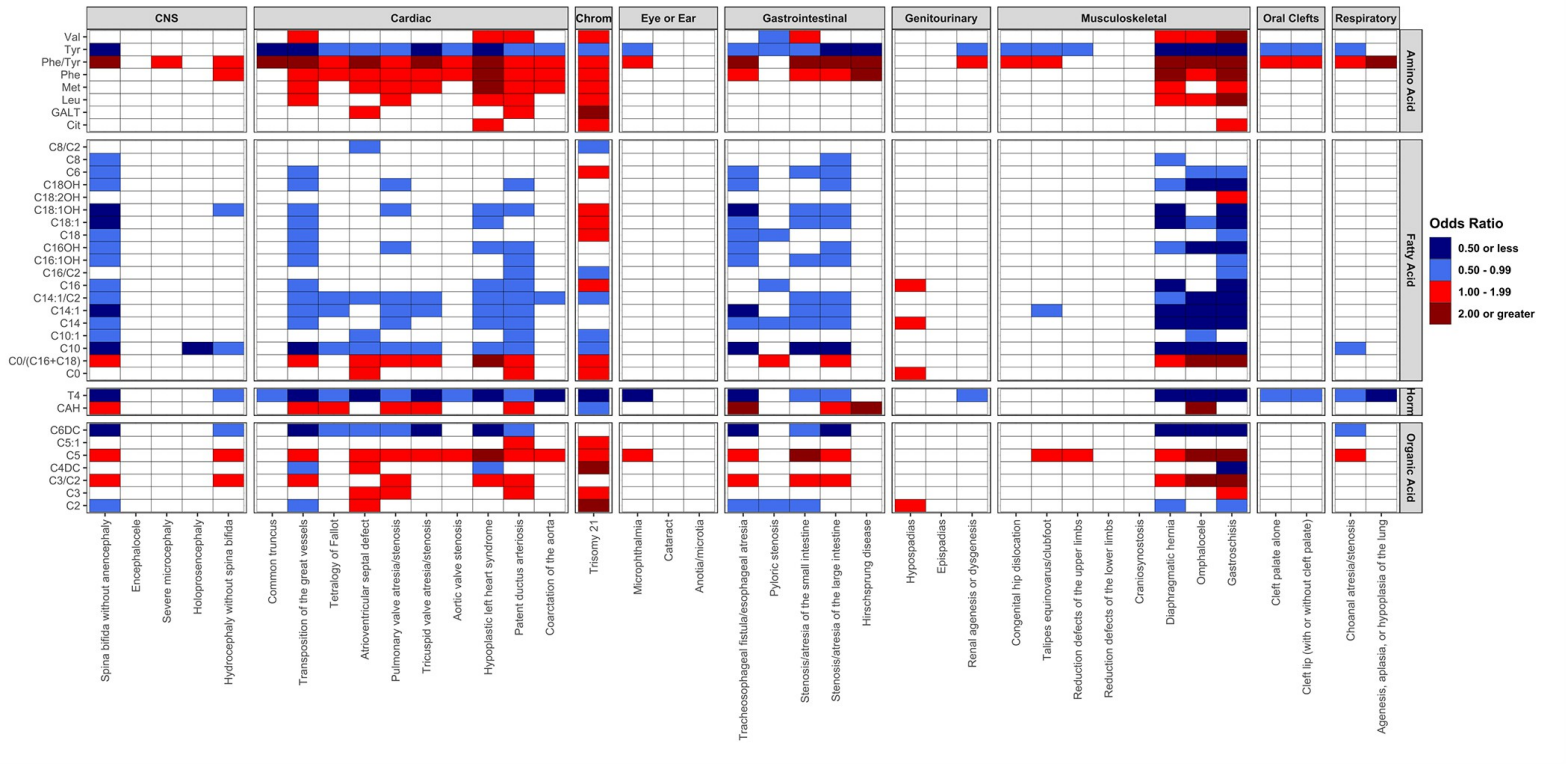
Heatmap of associations across all birth defects (N = 39) and newborn screening analytes (N = 36) based on discovery analysis. (Texas, 2007–2009).

Among the 1,404 associations evaluated (39 birth defects by 36 analytes), 418 associations (30%) were significant in the discovery analysis after Bonferroni correction (Fig 1). S4 Table presents all associations evaluated, including those non-significant. Next, 377 of the 418 associations (90%) were significant in the replication analysis. Single analytes most consistently associated with birth defects included the phenylalanine/tyrosine ratio (N = 29 birth defects), tyrosine (N = 28 birth defects), and thyroxine (N = 25 birth defects). Birth defects associated with more than 20 different analytes included gastroschisis (N = 29 analytes), several cardiovascular defects (e.g., transposition of the great vessels, N = 26 analytes), and spina bifida (N = 23 analytes).

Overall, associations were observed among several of the musculoskeletal defects, including diaphragmatic hernia, omphalocele, and gastroschisis (Fig 1). Generally, lower levels of fatty acid oxidation analytes and higher levels of amino acid analytes were associated with these anomalies. Associations across cardiac defects were also observed. For example, all cardiac defects were associated with higher a combination of phenylalanine/tyrosine ratios and lower levels of tyrosine (when considered as a single analyte). Trisomy 21 was associated with higher levels of all amino acids and lower levels of both hormonal analytes; trisomy 21 also showed positive associations with five out of the seven organic acids.

As noted, the finding most consistently associated with birth defects was the phenylalanine/tyrosine ratio. In fact, higher phenylalanine/tyrosine ratios were associated with 29 of the 39 birth defects evaluated including gastroschisis (replication OR = 11.36, 95% CI: [6.13, 21.05], p-value = 1.17×10^−14^) and transposition of the great vessels (replication OR = 3.37, 95% CI: [2.66, 4.27], p-value = 6.10×10^−24^).

The major structural birth defects with the most significant associations with NBS analytes included gastroschisis and transposition of the great vessels (Table 2). Gastroschisis was most significantly associated with tyrosine levels (replication OR = 0.28, 95% CI: [0.21, 0.36], p-value = 3.32×10^−20^). Meanwhile, transposition of the great vessels was most significantly associated with higher ratios of phenylalanine/tyrosine (replication OR = 3.37, 95% CI: [2.66, 4.27], p-value = 6.10×10^−24^) and lower levels of thyroxine (replication OR = 0.33, 95% CI: [0.26, 0.41], p-value = 6.82×10^−22^).

**Table 2:** Top 50 significant^a^ newborn screening analytes across all birth defects. (Texas, 2007–2009).

Birth Defect	Analyte	Discovery			Replication		
		P-value	trend (OR)	OR 95% CI	P-value	trend (OR)	OR 95% CI
Gastroschisis	Tyr	6.85E-56	0.21	(0.17, 0.25)	3.32E-20	0.28	(0.21, 0.36)
Transposition of the great vessels	Phe/Tyr	1.02E-46	2.92	(2.52, 3.38)	6.10E-24	3.37	(2.66, 4.27)
Gastroschisis	Phe/Tyr	1.54E-43	12.41	(8.69, 17.73)	1.17E-14	11.36	(6.13, 21.05)
Gastroschisis	C10	8.84E-42	0.15	(0.11, 0.20)	4.34E-17	0.15	(0.10, 0.24)
Gastroschisis	C3/C2	2.66E-41	3.19	(2.70, 3.78)	2.33E-14	2.61	(2.04, 3.33)
Stenosis/atresia of the large intestine	Phe/Tyr	4.54E-40	2.76	(2.37, 3.20)	2.80E-20	2.69	(2.18, 3.32)
Transposition of the great vessels	T4	1.74E-38	0.39	(0.34, 0.45)	6.82E-22	0.33	(0.26, 0.41)
Gastroschisis	C14:1	2.73E-38	0.24	(0.20, 0.30)	6.50E-17	0.14	(0.09, 0.23)
Transposition of the great vessels	Tyr	4.28E-37	0.45	(0.39, 0.51)	2.61E-16	0.48	(0.41, 0.58)
Gastroschisis	C14:1/C2	1.87E-36	0.33	(0.27, 0.39)	4.29E-17	0.28	(0.20, 0.37)
Gastroschisis	T4	6.53E-36	0.24	(0.19, 0.30)	3.46E-15	0.23	(0.16, 0.33)
Gastroschisis	C14	4.91E-35	0.36	(0.30, 0.42)	1.18E-17	0.23	(0.16, 0.32)
Stenosis/atresia of the large intestine	Tyr	1.90E-34	0.44	(0.38, 0.50)	2.01E-17	0.46	(0.38, 0.55)
Atrioventricular septal defect	Phe/Tyr	8.09E-33	2.32	(2.02, 2.66)	4.82E-17	2.72	(2.15, 3.44)
Gastroschisis	Val	1.57E-32	2.49	(2.14, 2.89)	8.04E-15	2.71	(2.10, 3.48)
Gastroschisis	C5	1.97E-32	3.21	(2.65, 3.89)	3.52E-13	3.18	(2.33, 4.35)
Stenosis/atresia of the small intestine	Phe/Tyr	4.41E-32	3.08	(2.56, 3.72)	1.89E-13	2.88	(2.17, 3.81)
Transposition of the great vessels	C6DC	6.50E-31	0.47	(0.42, 0.54)	1.00E-21	0.35	(0.28, 0.44)
Atrioventricular septal defect	T4	7.27E-31	0.41	(0.36, 0.48)	7.94E-11	0.50	(0.40, 0.61)
Gastroschisis	Leu	2.53E-30	2.35	(2.03, 2.72)	3.00E-12	2.32	(1.83, 2.95)
Spina bifida without anencephaly	C6DC	6.99E-30	0.37	(0.31, 0.43)	8.98E-16	0.34	(0.27, 0.45)
Spina bifida without anencephaly	Phe/Tyr	3.43E-29	2.49	(2.12, 2.92)	3.18E-14	2.54	(2.00, 3.24)
Transposition of the great vessels	C14:1	1.59E-28	0.52	(0.46, 0.58)	3.53E-12	0.55	(0.47, 0.65)
Transposition of the great vessels	C10	1.24E-27	0.52	(0.47, 0.59)	7.25E-15	0.50	(0.42, 0.59)
Coarctation of the aorta	Phe/Tyr	1.75E-27	1.90	(1.69, 2.13)	4.71E-09	1.64	(1.39, 1.93)
Spina bifida without anencephaly	C10	4.78E-25	0.45	(0.38, 0.52)	1.37E-15	0.35	(0.27, 0.45)
Stenosis/atresia of the small intestine	Tyr	1.12E-24	0.45	(0.39, 0.53)	5.47E-09	0.52	(0.41, 0.65)
Gastroschisis	C18:1	1.21E-24	0.49	(0.43, 0.56)	3.64E-11	0.44	(0.35, 0.56)
Tetralogy of Fallot	Phe/Tyr	4.68E-24	2.10	(1.82, 2.42)	6.75E-12	1.96	(1.61, 2.37)
Transposition of the great vessels	C14	5.45E-24	0.57	(0.51, 0.63)	1.17E-08	0.62	(0.53, 0.73)
Stenosis/atresia of the small intestine	C10	1.11E-23	0.41	(0.34, 0.49)	4.48E-09	0.46	(0.36, 0.6)
Coarctation of the aorta	T4	1.69E-23	0.55	(0.48, 0.61)	1.09E-15	0.45	(0.37, 0.54)
Transposition of the great vessels	C18:1	1.96E-23	0.56	(0.5, 0.63)	1.07E-07	0.64	(0.55, 0.76)
Stenosis/atresia of the large intestine	C10	2.23E-23	0.53	(0.47, 0.6)	1.27E-14	0.49	(0.40, 0.58)
Gastroschisis	C0/(C16+C18)	2.33E-23	2.20	(1.89, 2.57)	5.57E-12	2.63	(2.00, 3.47)
Spina bifida without anencephaly	Tyr	2.41E-23	0.48	(0.42, 0.56)	1.64E-12	0.45	(0.36, 0.56)
Pulmonary valve atresia/stenosis	Phe/Tyr	5.90E-23	1.46	(1.35, 1.57)	2.88E-18	1.66	(1.48, 1.86)
Spina bifida without anencephaly	C14:1	6.14E-23	0.47	(0.41, 0.55)	3.60E-14	0.41	(0.32, 0.51)
Hypoplastic left heart syndrome	Phe/Tyr	8.98E-23	4.86	(3.55, 6.67)	1.30E-09	3.87	(2.50, 5.98)
Cleft lip (with or without cleft palate)	Phe/Tyr	1.21E-22	1.46	(1.35, 1.57)	2.23E-06	1.31	(1.17, 1.46)
Gastroschisis	Phe	3.97E-22	1.96	(1.71, 2.25)	7.41E-10	2.03	(1.62, 2.53)
Cleft lip (with or without cleft palate)	Tyr	4.39E-22	0.69	(0.64, 0.74)	4.80E-05	0.80	(0.71, 0.89)
Pulmonary valve atresia/stenosis	T4	4.62E-22	0.67	(0.62, 0.73)	6.62E-14	0.63	(0.56, 0.71)
Diaphragmatic hernia	T4	7.34E-22	0.21	(0.15, 0.29)	3.60E-07	0.31	(0.2, 0.48)
Stenosis/atresia of the large intestine	C6DC	7.35E-22	0.53	(0.47, 0.6)	3.40E-17	0.42	(0.34, 0.51)
Gastroschisis	C4DC	8.22E-22	0.51	(0.44, 0.58)	5.85E-12	0.43	(0.34, 0.54)
Diaphragmatic hernia	Phe/Tyr	1.00E-21	3.15	(2.49, 3.99)	7.15E-08	3.53	(2.23, 5.59)
Tracheosophageal fistula/esophageal atresia	Phe/Tyr	2.46E-21	3.80	(2.89, 5.01)	2.71E-06	2.43	(1.67, 3.51)
Gastroschisis	Met	5.00E-21	1.84	(1.62, 2.09)	7.41E-07	1.65	(1.35, 2.01)
Spina bifida without anencephaly	T4	5.26E-21	0.48	(0.42, 0.56)	9.49E-14	0.40	(0.32, 0.51)

^a^ Bonferroni adjustment in discovery and at a nominal p<0.05 in replication

### 3.2 Isolated birth defects

**Fig 2:**
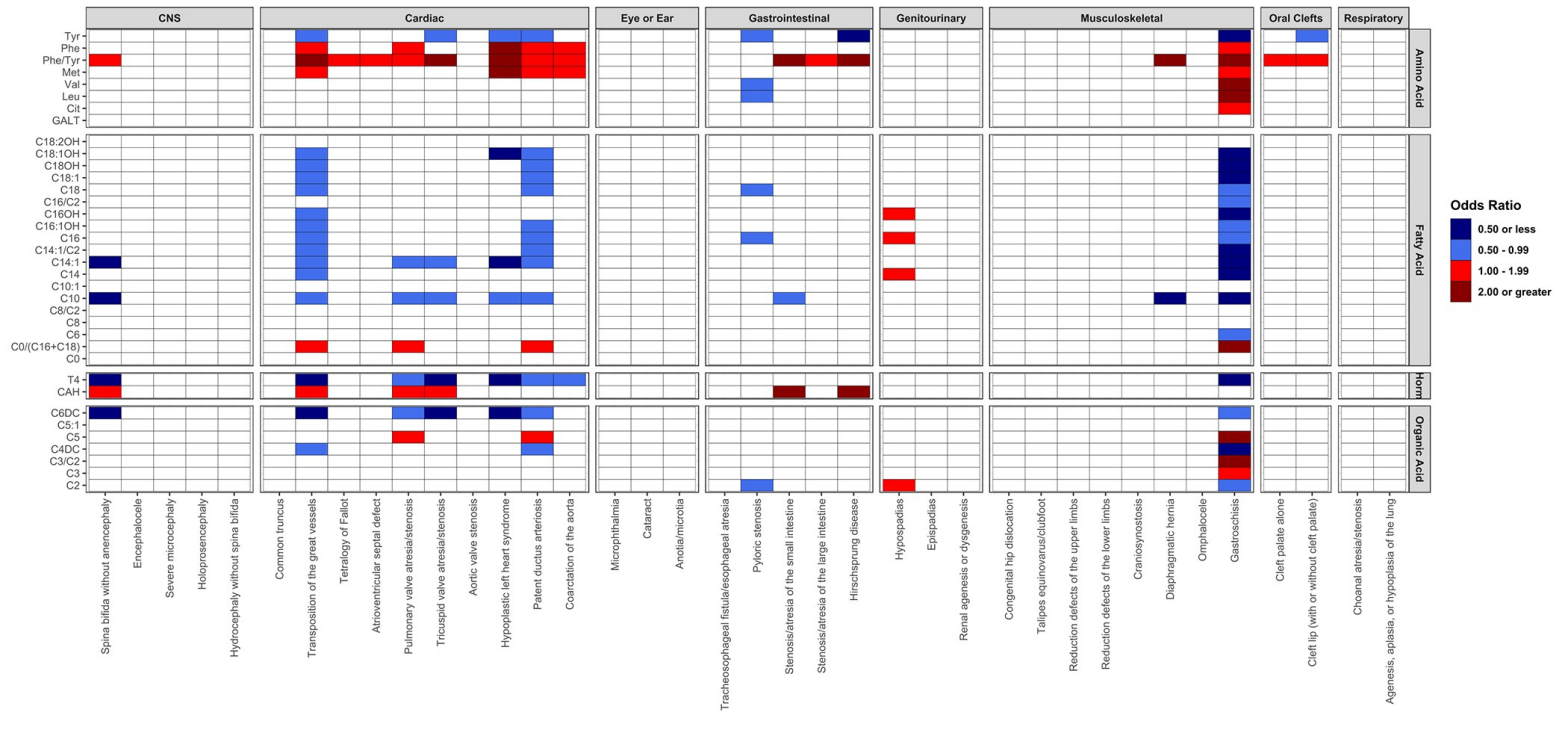
Heatmap of associations across all isolated birth defects (N = 38) and newborn screening analytes (N = 36) based on discovery analysis. (Texas, 2007–2009).

When restricting to children with isolated birth defects, there were fewer significant associations in the discovery analysis (N = 145, 10%) (Fig 2). However, the associations reported in the overall birth defect analysis (Table 2) for gastroschisis and transposition of the great vessels were also reported in the isolated birth defect analysis (Table 3). Specifically, 28 of the 36 analytes were significantly associated with isolated gastroschisis, with lower levels of tyrosine being the strongest observed association (replication OR = 0.26, 95% CI: [0.19, 0.37], p = 2.02×10^−15^). Twenty of the 36 analytes included in the analysis were significantly associated with transposition of the great vessels, with higher phenylalanine/tyrosine ratios being the most significant association (replication OR = 2.42, 95% CI: [1.82, 3.21], p = 1.28×10^−9^).

**Table 3:** Top 15 significant^a^ newborn screening analytes for isolated gastroschisis and isolated transposition of the great vessels. (Texas, 2007–2009).

Analyte	Discovery			Replication		
	P-value	trend (OR)	OR 95% CI	P-value	trend (OR)	OR 95% CI
Gastroschisis						
Tyr	4.42e-41	0.22	(0.17, 0.27)	2.02e-15	0.26	(0.19, 0.37)
C10	2.15e-30	0.14	(0.01, 0.19)	1.25e-11	0.13	(0.08, 0.24)
C3/C2	2.05e-29	3.09	(2.54, 3.76)	9.44e-12	2.96	(2.16, 4.04)
C14:1	8.22e-28	0.24	(0.19, 0.31)	5.96e-11	0.11	(0.05, 0.21)
Phe/Tyr	3.92e-27	17.40	(10.36, 29.24)	8.63e-13	5.78	(3.57, 9.35)
T4	1.07e-26	0.21	(0.16, 0.28)	2.25e-11	0.25	(0.17, 0.37)
C14:1/C2	3.58e-25	0.35	(0.28, 0.42)	3.55e-12	0.26	(0.18, 0.38)
C14	4.07e-25	0.36	(0.30, 0.44)	5.4e-12	0.23	(0.15, 0.35)
C5	1.06e-23	3.22	(2.56, 4.05)	1.05e-08	2.65	(1.90, 3.70)
Val	2.12e-23	2.48	(2.08, 2.97)	2.76e-10	2.55	(1.91, 3.41)
Leu	6.83e-21	2.29	(1.93, 2.72)	7e-09	2.23	(1.70, 2.93)
C18:1	2.16e-18	0.48	(0.41, 0.57)	1.23e-07	0.47	(0.35, 0.62)
C0/(C16+C18)	2.85e-18	2.36	(1.95, 2.87)	1.39e-07	2.24	(1.66, 3.02)
C4DC	2.44e-17	0.49	(0.42, 0.58)	1.17e-07	0.48	(0.37, 0.63)
C6DC	2.36e-16	0.48	(0.40, 0.57)	9.44e-06	0.56	(0.44, 0.73)
Transposition of the Great Vessels						
Phe/Tyr	3.92E-23	3.76	(2.90, 4.89)	1.28E-09	2.42	(1.82, 3.21)
T4	2.74E-19	0.36	(0.28, 0.45)	5.07E-09	0.44	(0.33, 0.58)
Tyr	4.38E-18	0.42	(0.34, 0.51)	1.38E-06	0.54	(0.42, 0.70)
C14	5.50E-17	0.46	(0.38, 0.55)	0.000516	0.66	(0.52, 0.84)
C10	1.44E-16	0.46	(0.38, 0.55)	5.04E-05	0.62	(0.49, 0.78)
C18:1	1.50E-16	0.45	(0.37, 0.54)	0.000204	0.64	(0.51, 0.81)
C14:1	2.33E-16	0.46	(0.39, 0.56)	0.000796	0.68	(0.54, 0.85)
C6DC	5.79E-16	0.45	(0.37, 0.54)	9.05E-08	0.49	(0.38, 0.64)
C16	9.18E-14	0.51	(0.43, 0.61)	0.000436	0.66	(0.52, 0.83)
C18	1.06E-13	0.51	(0.43, 0.61)	0.00487	0.72	(0.57, 0.9)
C16:1OH	1.53E-10	0.57	(0.48, 0.68)	0.0186	0.75	(0.59, 0.95)
C18:1OH	4.89E-10	0.53	(0.44, 0.65)	0.00121	0.64	(0.49, 0.84)
C2	6.77E-10	0.59	(0.50, 0.70)	0.0634	0.81	(0.65, 1.01)
Phe	3.57E-09	1.69	(1.42, 2.01)	1.44E-06	1.88	(1.45, 2.43)
C0/(C16+C18)	4.53E-09	1.66	(1.40, 1.97)	1.60E-06	1.82	(1.42, 2.32)

^a^ Bonferroni adjustment in discovery and at a nominal p<0.05 in replication

## 4 Discussion

In one of the largest studies on this topic, our findings suggest that several NBS analytes are associated with a range of structural birth defects. This is consistent with results from smaller studies suggesting that certain NBS analytes are associated with structural birth defects, such as craniosynostosis and choanal atresia [3, 4]. These results could inform our understanding of the etiologies of birth defects. Notable and consistent observations include: 1) associations between several NBS analyte levels and a wide range of cardiac defects; 2) associations between numerous NBS analytes and gastroschisis; 3) several analyte-spina bifida associations; and 4) the association between the phenylalanine/tyrosine ratio with many different birth defects (N = 29 birth defects).

There are several potential reasons underlying our observed associations, largely depending on the analyte-birth defect pattern in question. First, known or uncharacterized genetic syndromes may underlie some of the observations. Specifically, certain genetic syndromes are characterized by multiple congenital anomalies (MCAs) and have metabolic derangements. For example, cardiac, vertebral, and renal defects have been observed in nicotinamide adenine dinucleotide (NAD) deficiency, a disruption in NAD synthesis due to a genetic disorder [16]. While NAD deficiency is rare, there could be other uncharacterized genetic syndromes with features including MCAs and metabolic derangements. While we did not exclude cases with known genetic syndromes, we did evaluate children with isolated birth defects and still observed consistent and significant associations. Second, it is possible that altered analyte levels could be a consequence of birth defects (e.g., gastrointestinal defects could lead to altered metabolites). Third, some of the observed associations may suggest novel etiologic factors, which with further research, may provide insights into the pathways leading to birth defects.

We observed consistently strong associations across several NBS analytes and multiple cardiac defects. For example, many of the cardiac defects included were associated with higher ratios of phenylalanine/tyrosine and lower tyrosine levels. Specifically, transposition of the great vessels, one of the more common and severe congenital heart defects, was among the top five most significant reported association across all analyte-birth defect associations evaluated [17]. While the reasons underlying these associations are likely complex, previous work has demonstrated that increased maternal levels of phenylalanine (>30 mg/dL) during the first trimester are significantly associated with an increased risk of giving birth to a child with a congenital heart defect [18]. Similar trends have also been reported among women with phenylalanine levels exceeding 14 mg/dL [18]. With this in mind, it follows that the analyte associations we report may reflect novel insights to the etiologies of cardiac defects, where maternal phenylalanine levels might play a role in the development of cardiac defects. An association was seen between NBS analytes and patent ductus arteriosus. This defect is part of the normal transition from intrauterine to extrauterine life and is frequently associated with preterm birth. Abnormalities in NBS results can be seen in preterm infants, even in the absence of a birth defect.

Gastroschisis is a major structural birth defect, where we observed several strong and significant associations. These associations could implicate new etiologies for this clinically important condition. For example, studies suggest that maternal nutrition may play a role in the development of gastroschisis. Specifically, it has been reported that low serum fatty acid levels are observed in women who give birth to children with gastroschisis [19]. In our study, lower levels of 13 of the 19 fatty acid NBS analytes were associated with an increased risk for gastroschisis, suggesting that the lower levels may lead to gastroschisis. Alternatively, our results may suggest that gastroschisis leads to disrupted pathways causing altered levels of analytes since children with gastroschisis do not receive adequate *in utero* nutrition. Notably, omphalocele did not show consistent significant associations with NBS analytes, highlighting the potential impact of having an open abdominal wall on analyte changes secondary to the defect (i.e., reverse causality).

Also of interest, several NBS analytes were associated with spina bifida. Specifically, we observed that several fatty acids, hormonal analytes, tyrosine, and phenylalanine/tyrosine were strongly and consistently associated with spina bifida. It is worth noting that some observed associations may be related to the development of this condition. For example, it has been suggested that placental transport of amino acids might be depressed in neural tube defects, such as spina bifida, which may, in part, explain our observation of an inverse association between lower levels of tyrosine and risk for spina bifida [20]. Previous studies have observed associations with several metabolites in maternal plasma and amniotic fluid associated with the development of spina bifida [21]. For example, deficiencies in glucose, carnitine, and amino acids observed in the amniotic fluid may contribute to spina bifida pathogenesis. Further work is needed to evaluate how these metabolites may interact with NBS analytes to better understand the etiology of spina bifida. Ultimately, improved understanding of the etiology of birth defects such as spina bifida could lead to better screening and primary prevention of these important defects.

Abnormal phenylalanine/tyrosine ratios were significantly associated with 29 birth defects in our study. Phenylalanine/tyrosine is a ratio of two amino acids and is an important indicator of metabolic control. Phenylalanine is an essential α amino acid that ultimately makes tyrosine, an essential component for production of several brain chemicals such as epinephrine, norepinephrine, and dopamine [18]. Phenylketonuria (PKU) is a metabolic disease with known genetic etiology that prevents the body from breaking down phenylalanine, leading to a build-up of phenylalanine and, in turn, a tyrosine deficiency. Maternal PKU refers to the teratogenic effects of PKU during pregnancy [22]. Maternal PKU has been associated with intellectual disability, microcephaly, and congenital heart defects [22]. Notably, tyrosine levels are necessary to support fetal development [23]. Based on that, a tyrosine-deficient environment could lead to structural birth defects [23]. A dose-response relationship has been reported where lower frequencies of birth defects are observed at lower phenylalanine levels [24]. High levels of phenylalanine leading to lower tyrosine levels, even in women without PKU, may lead to metabolic changes and thus the onset of birth defects during gestation [23, 24]. Taken together, the results from our study as well as the previous studies could indicate a potential role of phenylalanine/tyrosine ratio in the onset of several birth defects. With further replication, these results could point to the utility of the phenylalanine/tyrosine ratio as an important screening tool for these associated birth defects, especially those not apparent at birth.

Strengths of our study include our large population-based cohort and agnostic analytic approach. Additionally, we attempted to statistically replicate our findings by creating discovery and replication sets during analysis [15]. Finally, our results remained consistent in a sensitivity analysis meant to evaluate the potential impact of having multiple vs. isolated birth defects.

However, our study should be considered in light of several limitations. First, some associations may have been (e.g., with cardiac defects) due to undiagnosed syndromes, though our sub-analysis of cases with isolated defects likely addressed this potential concern. Second, reverse causality may have been present, whereby altered analyte levels were a consequence of the condition (e.g., gastroschisis), or of the care provided to the baby because of the defect, which could alter timing of specimen collection or the food intake before specimen collection. Of note, NBS analytes may differ based on total parenteral nutrition (TPN). However, we had sufficient data to remove the cases to prevent any bias this confounder may present. Third, it is possible that differences in analyte levels might be due to systematic differences in when samples were collected, especially for those infants admitted to the NICU. However, we attempted to lessen the impact of these potential differences by selecting later screens and adjusting for factors like gestational age. It is notable that the Bonferroni correction for multiple testing has been criticized for possibly being overly conservative. However, given the large number of tests performed, we opted for a stricter criterion to focus our results and interpretations on the strongest associations observed. That said, the findings of this study are still important as they point to the need for genomic and metabolomic profiling to understand the causes and consequences of metabolic derangements in children with birth defects.

Overall, we have demonstrated in our large population-based assessment that a range of NBS analytes are associated with a spectrum of structural birth defects. Importantly, this study was the first to take an untargeted approach to identify associations across a range of NBS analytes and birth defects. Further studies are necessary to replicate our findings and examine the potential mechanisms underlying some of our observed associations. Ultimately, the results of this study could help guide the establishment of screening for birth defects that are not always apparent at birth, as well as point to new strategies for diagnostic and supportive care for these children.

## Supporting information

S1 Table Included birth defects. (DOCX)

S2 Table Presentation of 39 birth defects and the respective categorizations included in analyses. (DOCX)

S3 Table Descriptive table presenting the 36 newborn screening analytes included in analyses. (DOCX)

S4 Table All birth defect-analyte association results. (XLSX)

S1 File NBS protocol. (DOC)
