# Supplementary material for: Newborn screening analytes and structural birth defects among 27,000 newborns
Source: PLoS One. 2024 Jul 5;19(7):e0304238. doi: 10.1371/journal.pone.0304238 (PMC11226011; doi:10.1371/journal.pone.0304238)
Supplement: S1 Table — (DOCX) [file pone.0304238.s001.docx]

**S1 Table. Included birth defects**

| **Birth Defect Category** | **Birth Defect** |
| --- | --- |
| Central Nervous System | Spina bifida without anencephaly |
|  | Encephalocele |
|  | Microcephaly, severe (head circumference <3rd percentile) |
|  | Holoprosencephaly |
|  | Hydrocephaly without spina bifida |
| Eye or Ear | Microphthalmia |
|  | Cataract |
|  | Anotia/microtia |
| Cardiac | Common truncus |
|  | Transposition of the great vessels |
|  | Tetralogy of Fallot |
|  | Atrioventricular septal defect (endocardial cushion defect) |
|  | Pulmonary valve atresia or stenosis |
|  | Tricuspid valve atresia or stenosis |
|  | Aortic valve stenosis |
|  | Hypoplastic left heart syndrome |
|  | Patent ductus arteriosus |
|  | Coarctation of the aorta |
| Respiratory | Choanal atresia or stenosis |
|  | Agenesis, aplasia, or hypoplasia of the lung |
| Oral Clefts | Cleft palate alone |
|  | Cleft lip with or without cleft palate |
| Gastrointestinal | Tracheosophageal fistula/esophageal atresia |
|  | Pyloric stenosis |
|  | Stenosis or atresia of the small intestine |
|  | Stenosis or atresisa of the large intestine |
|  | Hirschsprung disease |
| Genitourinary | Hypospadias (cases and prevalence among males) |
|  | Epispadias |
|  | Renal agenesis or dysgenesis |
| Musculoskeletal | Congenital hip dislocation |
|  | Talipes equinovarus/clubfoot |
|  | Reduction defects of the upper limbs |
|  | Reduction defects of the lower limbs |
|  | Craniosynostosis |
|  | Diaphragmatic hernia |
|  | Omphalocele |
|  | Gastroschisis |
| Chromosomal | Trisomy 21 |

| **Analyte Type** | **Name for Analyte or Analyte Ratio** | **Analyte Abbreviation** |
| --- | --- | --- |
| Hormonal | 17-Hydroxyprogesterone | CAH |
|  | Thyroxine | T4 |
| Metabolic: Amino Acids | Citrulline | Cit |
|  | Galactose-1-phosphate uridylphosphorase | GALT |
|  | Leucine/isoleucine | Leu |
|  | Methionine | Met |
|  | Phenylalanine | Phe |
|  | Phenylalanine/tyrosine | Phe/Tyr |
|  | Tyrosine | Tyr |
|  | Valine | Val |
| Metabolic: Fatty Acid Oxidation | Free carnitine | CO |
|  | Free carnitine/(hexadecanoylcarnitine+octadecanoylcarnitine) | CO/(C16 + C18) |
|  | Hexanoylcarnitine | C6 |
|  | Ocatnoylcarinitine | C8 |
|  | Octanoylcarnitine/acetylcarnitine | C8/C2 |
|  | Decanoylcarnitine | C10 |
|  | Decenoylcarnitine | C10:1 |
|  | Tetradecanoylcarnitine | C14 |
|  | Tetradecenoylcarnitine | C14:1 |
|  | Tetradecenoylcarnitine/acetylcarnitine | C14:1/C2 |
|  | Hexadecanoylcarnitine | C16 |
|  | Hydroxy-hexadecanoylcarnitine | C16-0H |
|  | 3-Hydroxy-hexadecanoylcarnitine | C16:1-OH |
|  | Hexadecanoylcarnitine/acetylcarnitine | C16/C2 |
|  | Octadecanoylcarnitine | C18 |
|  | Octodecenoylcarnitine | C18:1 |
|  | 3-Hydroxy-octadecanoylcarnitine | C18-OH |
|  | Hydroxy-octadecenoylcarnitine | C18:1-OH |
|  | 3-Hydroxy-linoleylcarnitine | C18:2-OH |
| Metabolic: Organic Acids | Acetylcarnitine | C2 |
|  | Propionylcarnitine | C3 |
|  | Propionylcarnitine/Acetylcarnitine | C3/C2 |
|  | Methylmalonylcarnitine | C4DC |
|  | Isovalerylcarnitine | C5 |
|  | Tiglylcarnitine | C5:1 |
|  | Adipylcarnitine | C6DC |
