# Supplementary material for: Newborn screening analytes and structural birth defects among 27,000 newborns
Source: PLoS One. 2024 Jul 5;19(7):e0304238. doi: 10.1371/journal.pone.0304238 (PMC11226011; doi:10.1371/journal.pone.0304238)
Supplement: S2 Table — (DOCX) [file pone.0304238.s002.docx]

**S2 Table. Presentation of 39 birth defects and the respective categorizations included in analyses.**

| **Birth Defect Category** | **Birth Defect** | **BPA Codes** | **Case Count** |
| --- | --- | --- | --- |
| Central Nervous System | Spina bifida without anencephaly | 741.000–741.990, without 740.000–740.100 | 327 |
|  | Encephalocele | 742.000–742.090 | 53 |
|  | Microcephaly, severe (head circumference <3rd percentile) | 742.100, 742.486 | 390 |
|  | Holoprosencephaly | 742.26 | 65 |
|  | Hydrocephaly without spina bifida | 742.300–742.380 and 742.390, without 741.000-741.990 | 655 |
| Eye or Ear | Microphthalmia | 743.1 | 262 |
|  | Cataract | 743.320, 743.325, 743.326 | 208 |
|  | Anotia/microtia | 744.010, 744.210 | 369 |
| Cardiac | Common truncus | 745.000–745.010 | 74 |
|  | Transposition of the great vessels | 745.100–745.120, 745.180, 745.190 | 490 |
|  | Tetralogy of Fallot | 745.200, 746.840 | 376 |
|  | Atrioventricular septal defect (endocardial cushion defect) | 745.600–745.690 | 417 |
|  | Pulmonary valve atresia or stenosis | 746.000–746.010 | 1036 |
|  | Tricuspid valve atresia or stenosis | 746.100, 746.106 | 171 |
|  | Aortic valve stenosis | 746.300 | 238 |
|  | Hypoplastic left heart syndrome | 746.700 | 185 |
|  | Patent ductus arteriosus | 747.000 | 6212 |
|  | Coarctation of the aorta | 747.100–747.190 | 482 |
| Respiratory | Choanal atresia or stenosis | 748.000 | 125 |
|  | Agenesis, aplasia, or hypoplasia of the lung | 748.500–748.510 | 90 |
| Oral Clefts | Cleft palate alone | 749.000–749.090 | 615 |
|  | Cleft lip with or without cleft palate | 749.100–749.220 | 1009 |
| Gastrointestinal | Tracheosophageal fistula/esophageal atresia | 750.300–750.350 | 181 |
|  | Pyloric stenosis | 750.510 | 2066 |
|  | Stenosis or atresia of the small intestine | 751.100–751.195 | 317 |
|  | Stenosis or atresisa of the large intestine | 751.200–751.240 | 452 |
|  | Hirschsprung disease | 751.300–751.340 | 148 |
| Genitourinary | Hypospadias (cases and prevalence among males) | 752.600–752.607, 752.620, 752.625–752.627 | 3177 |
|  | Epispadias | 752.610 | 108 |
|  | Renal agenesis or dysgenesis | 753.000–753.010 | 500 |
| Musculoskeletal | Congenital hip dislocation | 754.300 | 450 |
|  | Talipes equinovarus/clubfoot | 754.500, 754.730 | 1489 |
|  | Reduction defects of the upper limbs | 755.200–755.290 | 382 |
|  | Reduction defects of the lower limbs | 755.300–755.390 | 165 |
|  | Craniosynostosis | 756.000–756.030 | 571 |
|  | Diaphragmatic hernia | 756.610–756.617 | 181 |
|  | Omphalocele | 756.700 | 125 |
|  | Gastroschisis | 756.710 | 526 |
| Chromosomal | Trisomy 21 | 758.000–758.090 | 1266 |
