# Supplementary material for: Newborn screening analytes and structural birth defects among 27,000 newborns
Source: PLoS One. 2024 Jul 5;19(7):e0304238. doi: 10.1371/journal.pone.0304238 (PMC11226011; doi:10.1371/journal.pone.0304238)
Supplement: S3 Table — (DOCX) [file pone.0304238.s003.docx]

**S3 Table. Descriptive table presenting the 36 newborn screening analytes included in analyses.**

| **Analyte Type** | **Name for Analyte or Analyte Ratio** | **Analyte Abbreviation** |
| --- | --- | --- |
| Hormonal | 17-Hydroxyprogesterone | CAH |
|  | Thyroxine | T4 |
| Metabolic: Amino Acids | Citrulline | Cit |
|  | Galactose-1-phosphate uridylphosphorase | GALT |
|  | Leucine/isoleucine | Leu |
|  | Methionine | Met |
|  | Phenylalanine | Phe |
|  | Phenylalanine/tyrosine | Phe/Tyr |
|  | Tyrosine | Tyr |
|  | Valine | Val |
| Metabolic: Fatty Acid Oxidation | Free carnitine | CO |
|  | Free carnitine/(hexadecanoylcarnitine+octadecanoylcarnitine) | CO/(C16 + C18) |
|  | Hexanoylcarnitine | C6 |
|  | Ocatnoylcarinitine | C8 |
|  | Octanoylcarnitine/acetylcarnitine | C8/C2 |
|  | Decanoylcarnitine | C10 |
|  | Decenoylcarnitine | C10:1 |
|  | Tetradecanoylcarnitine | C14 |
|  | Tetradecenoylcarnitine | C14:1 |
|  | Tetradecenoylcarnitine/acetylcarnitine | C14:1/C2 |
|  | Hexadecanoylcarnitine | C16 |
|  | Hydroxy-hexadecanoylcarnitine | C16-0H |
|  | 3-Hydroxy-hexadecanoylcarnitine | C16:1-OH |
|  | Hexadecanoylcarnitine/acetylcarnitine | C16/C2 |
|  | Octadecanoylcarnitine | C18 |
|  | Octodecenoylcarnitine | C18:1 |
|  | 3-Hydroxy-octadecanoylcarnitine | C18-OH |
|  | Hydroxy-octadecenoylcarnitine | C18:1-OH |
|  | 3-Hydroxy-linoleylcarnitine | C18:2-OH |
| Metabolic: Organic Acids | Acetylcarnitine | C2 |
|  | Propionylcarnitine | C3 |
|  | Propionylcarnitine/Acetylcarnitine | C3/C2 |
|  | Methylmalonylcarnitine | C4DC |
|  | Isovalerylcarnitine | C5 |
|  | Tiglylcarnitine | C5:1 |
|  | Adipylcarnitine | C6DC |
