# Supplementary material for: Newborn screening analytes and structural birth defects among 27,000 newborns
Source: PLoS One. 2024 Jul 5;19(7):e0304238. doi: 10.1371/journal.pone.0304238 (PMC11226011; doi:10.1371/journal.pone.0304238)
Supplement: S1 File — (DOC) [file pone.0304238.s005.doc]

**S1 File. NBS Protocol**

**Assessing Newborn Screening Analytes, Environmental Toxicants and Birth Defects**

**A. Summary/Synopsis**

All data are held by the Texas Birth Defects Epidemiology and Surveillance (BDES) Branch. Data requests can be directed to birthdefects@dshs.texas.gov.

Birth defects are the leading cause of infant mortality in the U.S., and more than 65% are of unknown origin. Recent studies suggest maternal and infant biochemical profiles may be associated with certain birth defects. In addition, there is growing evidence that environmental toxicants may influence biochemical levels in pregnant women and infants. Because of this, we propose to 1) examine the association between environmental toxicants (e.g., hazardous air pollutants) and newborn screening analyte levels in Texas and 2) determine the relationship between newborn screening analyte levels and selected birth defects. We will use linked data from the Texas Department of State Health Services (DSHS) Laboratory’s newborn screening database, the DSHS Office of Program Decision and Support (Family and Community Health Division), the Texas Birth Defects Registry (TBDR) and the Texas Commission on Environmental Quality air monitoring network.

We are in a unique position to conduct these studies because 1) Texas has a well-established Newborn Screening Program, testing approximately 800,000 newborn specimens annually, 2) the Texas Birth Defects Registry is one of the largest population-based, active surveillance systems of birth defects in the U.S., 3) Texas has one of the most extensive air monitoring networks in the U.S., 4) we have assembled a strong group of research collaborators who have wide-ranging experience using these data sources and 5) experts at the Texas Department of State Health Services will conduct data linkages and provide additional input regarding analyses.

**B. Research Plan**

***1. Introduction/Background*** – Birth defects affect approximately 6% of births worldwide (1). In the U.S., birth defects are the leading cause of pediatric hospitalizations (2), medical expenditures (3) and death in the first year of life (4). Globally, at least 3.3 million children under the age of 5 die each year because of a birth defect (1). Although these outcomes are prevalent and clinically significant, little is known about their etiology.

It is believed that maternal and infant biochemical profiles (e.g., thyroid hormones, amino acids, galactose, methionine) may be associated with several birth defects and other adverse reproductive outcomes (5). Currently, newborn screening programs throughout the U.S. identify single gene disorders, based on extreme biochemical profiles (6). However, there is growing evidence that variation in these biochemical profiles may also influence a range of other birth defects.

Thyroid hormone levels are assessed in newborn screening to identify infants with extreme values indicative of congenital thyroid disease. There is also evidence that less extreme levels may influence the risk of other conditions (7). A few studies have indicated maternal thyroid hormones (using maternal thyroid disease and thyroid medication use as a proxy) may be associated with a variety of birth defects including craniosynostosis (8), central nervous system defects (9, 10, 11), limb deficiencies (10), hypospadias (9, 11) and choanal atresia (12). Infant thyroid hormone levels also appear to play an important role in development. Congenital hypothyroidism is associated with certain birth defects and impaired neurological function in childhood (reviewed in (13)). In the single published study assessing the association between infant thyroid hormone levels from newborn screening results and birth defects, low thyroid hormone levels were associated with cardiac defects (7). However, this study was based on data on 344 infants and remains to be confirmed.

Identifying the determinants of biochemical profiles is important in understanding the potential mechanism of disease. Although genetic factors most certainly play a role (6, 14), there is a growing amount of research indicating that environmental factors are also important determinants of biochemical phenotypes (14, 15). In particular, several lines of evidence indicate that environmental toxicants may act as endocrine disruptors.

Endocrine disruptors are chemicals that influence hormonal activity (16). Recent research has focused on the endocrine disrupting properties of environmental toxicants. Many chemicals found in the environment have been shown to influence hormone levels in animal models (17). It is believed that pregnant women and infants are particularly susceptible to the potential effects of these chemicals (14). One group of chemicals that are likely endocrine disruptors is hazardous air pollutants (HAPs) (15, 17). The U.S. Clean Air Act defines HAPs as mutagenic substances that may be responsible for adverse health effects. Texas ranks number one in the U.S. for HAPs concentrations, however, there is a great deal of spatial and temporal variability in levels across the state (18, 19). This makes Texas an ideal locale for studying the effects of HAPs on newborn analytes. Furthermore, this is an opportunity to study the effect of HAPs on other biochemical analytes that have not been previously assessed. In order to conduct this study, a strong group of collaborators has been assembled who have extensive experience with these data.

***2. Objectives/Specific Aims*** – Birth defects are somewhat prevalent and clinically severe, however, strategies for reducing the public health impact of these conditions are limited. As there are few clues regarding the causes of these outcomes, new studies assessing previously unexplored factors as they relate to endocrine disruption, and ultimately birth defects, are strongly warranted. The objective of this study is to assess not only how environmental toxicants affect newborn analyte levels, but also to examine how newborn analyte levels may be related to selected birth defects. In order to accomplish this objective we propose two aims:

**Aim 1 Determine if high concentrations of hazardous air pollutants found in the environment are associated with extreme levels of newborn screening analytes.**

Publically available data on environmental levels of hazardous air pollutants (HAPs) in Texas, collected by the Texas Commission on Environmental Quality air monitoring network and obtained from the U.S. Environmental Protection Agency (EPA), will be linked with results from the Texas Newborn Screening Program in order to determine the association between HAP concentrations and newborn analyte levels.

Data from birth certificates and newborn screening results from 2008 (linked by the Texas Department of State Health Services, Division of Family and Community Health Services) will be used for these analyses.

**Aim 2 Ascertain the relationship between newborn screening analyte values and selected birth defects.**

Data for live-born babies with birth defects in the TBDR will be linked with the DSHS Laboratory’s newborn screening records, as well as live birth records. The time period for this project is mid-2004 (when the lab database was established) through 2009. The linked data will be used to see if there is an association between newborn analyte levels and birth defects.

All data linkages between the Texas Birth Defects Registry and laboratory and vital records will be performed by individuals at the Texas Department of State Health Services.

**C. Subject Selection**

***1. Study Population*** – For Aim 1, subjects will be births in the state of Texas for the period specified in Section C2. Additional information on eligibility and ineligibility criteria are presented in Section C2 and C3.

For Aim 2, Cases will be drawn from the TBDR. The TBDR is maintained by the Birth Defects Epidemiology and Surveillance Branch of DSHS (20). The registry has actively monitored births, fetal deaths, and terminations in selected regions of Texas since 1996 and throughout the state since 1999. The TBDR is a population-based, active surveillance system. TBDR staff routinely visit hospitals and birthing centers, reviewing medical records to ascertain potential cases. Information is abstracted from medical records and held in strict confidence according to state and federal privacy laws. A random sample of controls (i.e., unaffected births) will be provided by the TBDR at a ratio of 4 controls to 1 case based on the largest case group. Additional information on eligibility and ineligibility criteria are presented in Section C2 and C3.

***Power:*** For Aim 1, with the proposed study population and mean endocrine levels reported in a previous study (21); we will be able to reject the null hypothesis that the population means of a high and low exposed group is equal with probability (power) 0.99. The Type I error probability associated with this test of this null hypothesis is 0.05. For Aim 2, as multiple case groups (and subgroups) will be assessed, a range of case group sizes and minimum detectable odds ratios are presented in Table 1. For our power calculations, exposure is binomial with 25% exposed (i.e., the upper quartile of exposure). These are reasonable effect sizes for the associates that are being evaluated. All power calculations were conducted using PS version 3.0 (22).

Table 1. Minimum detectable odds ratios for the association between newborn screening analytes and selected birth defects

|  | Power | | | | |
| --- | --- | --- | --- | --- | --- |
| Minimum Detectable Odds Ratio | *N* = 100 | *N* = 250 | *N* = 500 | *N* = 1,000 | *N* = 2,000 |
| 1.2 | 0.12 | 0.23 | 0.39 | 0.65 | 0.82 |
| 1.4 | 0.30 | 0.61 | 0.88 | 0.99 | 0.99 |
| 1.6 | 0.52 | 0.88 | 0.99 | 0.99 | 0.99 |
| 1.8 | 0.72 | 0.98 | 0.99 | 0.99 | 0.99 |
| 2.0 | 0.85 | 0.99 | 0.99 | 0.99 | 0.99 |

***2. Eligibility Criteria*** – For Aim 1, subjects will be eligible if they are unaffected (i.e., not born with a birth defect or a condition assessed by newborn screening, e.g., congenital hypothyroidism) and were born in 2008. The year 2008 was selected due to availability of linked birth certificate data with newborn screening results.

For Aim 2, eligible case groups include: 1) central nervous system defects, 2) eye and ear defects, 3) cardiac and circulatory defects, 4) respiratory defects, 5) oral clefts, 6) gastrointestinal defects, 7) genitourinary defects and 8) musculoskeletal defects. Only liveborn cases are eligible. Eligible controls will be unaffected births (i.e., those subjects from Aim 1). Eligible birth years are 2004 through 2009.

***3. Ineligibility Criteria*** – For Aim 1, subjects are ineligible if they were born prior to 2008 and if they do not have newborn screening results linked to birth certificates. Any baby receiving a blood transfusion prior to screening will be ineligible.

For Aim 2, case subjects are ineligible if they have a known syndrome (e.g., cases with chromosomal abnormalities will be excluded). Controls are ineligible if they have a birth defect or a condition assessed by newborn screening. Cases and controls born prior to 2004 are ineligible as newborn screening results are not available for this time period. As in Aim 1, any baby receiving a blood transfusion prior to screening will be ineligible.

***4. Recruitment/Registration*** – No subjects will be contacted for this study. No further recruitment is planned, and there is no follow-back component to this study.

**D. Protocol Details**

***1. Research Design and Methods*** – The studies for this project will be population-based and observational in scope. The research design for Aim 1 will be a retrospective cohort study in which an exposed group (i.e., those who are exposed to high concentrations of HAPs) will be compared to an unexposed group (i.e., those who are exposed to low concentrations of HAPs) in order to determine if exposure is associated with extreme biochemical analyte levels. Linear regression will be used to assess differences in mean levels of analytes between groups.

The research design for Aim 2 will be a case-control study in which cases (those with selected birth defects) will be compared to controls (unaffected births) in order to determine if biochemical analyte levels are associated with case status.

***2. Subject Assessment*** – No subjects will be contacted or directly assessed in this study. All information will be obtained from secondary data sources (e.g., vital records). The study populations are described in Section C1.

***3. Data*** – Request forms for birth certificate data and data from the TBDR, along with a list of newborn screening analytes requested for analysis, can be found in Appendix A. We are aware that two blood spots are taken at birth for newborn screening. A decision on which value to use will be made based upon availability and appropriate feedback by the Laboratory Services Section.

Standard demographic and clinical data fields are requested including: date of birth, birth weight, maternal age, race/ethnicity, education, tobacco use and medical risk factors for pregnancy. For Aim 1, latitude and longitude based on mother’s street address is requested in order to assign air pollutant measurements. This is the standard approach used in air pollution exposure assessment methodology (23, 24, 25) and will be important in determining if HAPs influence analyte levels. Furthermore, the principal investigator has previously obtained permission to use these data (latitude and longitude based on maternal address at delivery) (IRB# 08-037, 08-039 and 08-040) for studies assessing the association between HAPs and neural tube defects and published the results in aggregate (26). For Aim 2, all data will be de-identified.

All data linkages have been or will be conducted by DSHS, using personal identifiers (e.g., name). This will eliminate the need by collaborators external to DSHS to request data fields that include these personal identifiers. For Aim 1, newborn screening results for 2008 have already been linked to birth certificates by the Office of Program Decision and Support, Division of Community and Family Health Services. Data linkages between the Laboratory Services Section and TBDR (from mid-2004 onward) are underway, but to a limited extent, by the Texas Birth Defects Epidemiology and Surveillance Branch. As this is a resource-intensive process, for Aim 2, we will start with specific birth defect groups (e.g., craniosynostosis, choanal atresia and genitourinary defects) and the conventional newborn screening disorders (prior to NBS expansion in December 2006), but will expand the analyses to other defect and analyte groups as more data and linkage capacity become available.

The confidentiality of all participants in the proposed study will be fully protected. The principal investigator has completed the CITI Collaborative Institutional Training Initiative for Human Research (see Appendix B). Although latitude and longitude based on maternal address at delivery will be used (and kept strictly confidential) no other personal identifiers are being requested, and no attempt will be made to identify or locate study subjects using the latitude/longitude data. All study results will be reported only in the aggregate. Further, the computer files will be password protected to ensure database security, and the data will be destroyed upon completion of project. HIPAA policies and procedures will be followed throughout.

Hazardous air pollutant (HAP) levels (i.e., exposure data for Aim 1) are collected by the Texas Commission on Environmental Quality (TCEQ) and will be obtained from the EPA. The TCEQ is responsible for monitoring air quality in Texas and reporting ambient concentrations of air pollutants to the EPA’s Air Quality System (AQS). The AQS includes data for all HAPs monitored in Texas. Location of monitoring site, sample data, sampling duration and collection frequency along with other important variables are reported. The data are publicly available through the EPA. The EPA works with the TCEQ to conduct accuracy and precision checks. Generally, air samples are collected by pressurized or sub-atmospheric canisters and analyzed by gas chromatography. The typical sampling duration is 24 hours with a collection frequency of every 6th day.

***4. Statistical Design and Analysis*** – For Aim 1, a geographic information systems (GIS) approach will be used for exposure assessment. Specifically, inverse-distance interpolation will be used to estimate exposure based on latitude and longitude of maternal address at delivery (27). With inverse-distance weighting, relative weights diminish as the distance from the monitoring site increases. The inverse-distance interpolator is a weighted average of neighboring values (27). In this case, the weight assigned to each value is a function of the distance between a monitoring station and maternal address.

Linear regression will be used to determine the association between maternal exposure to HAPs (high versus low) and infant analyte levels. Potential covariates include: infant sex and maternal age, diabetes, tobacco use, education and race/ethnicity (obtained from birth certificates). Single and co-pollutant models will be used to examine confounding due to additional air pollutants. All covariates will be assessed by evaluating whether the regression coefficient indicating the association between maternal HAP concentrations and infant analyte levels changes by more than 10% when a potential confounder is included in the model. This strategy will result in a final model.

For Aim 2, logistic regression will be used to determine the association between infant analyte levels and selected birth defects. To examine the analyte-birth defect relationship, analyte levels will be categorized by quintiles among the controls. Other categories of exposure may also be assessed (e.g., low or reference exposure [< 25th percentile], medium exposure [25th–74th percentiles], high exposure [75th–89th percentiles], and very high exposure [90th percentile]) based on sample size. The exposure metric will also be assessed as a continuous variable. As in Aim 1, potential covariates include: infant sex and maternal age, diabetes, tobacco use, education and race/ethnicity. All covariates will be assessed by evaluating whether the odds ratio indicating the association between infant analyte levels and selected birth defects changes by more than 10% when a potential confounder is included in the model. This strategy will result in a final model for each birth defect included in this study (see Section C2 for a list of selected birth defect groups).

As a first step for Aim 2, due to the time and effort required for linking newborn screening data, the following analytes and birth defects will be assessed initially: thyroxine (T4) and thyroid-stimulating hormone (TSH) and craniosynostosis; T4/TSH and choanal atresia; 17-hydroxy progesterone and genitourinary defects. When Registry data for 2008 or 2009 deliveries are complete, we will look at the association of selected additional birth defects with NBS analyte values, such as spina bifida and methionine (the analyte of homocystinuria, one of the “new” disorders for which screening was implemented in December 2006).

All analyses will be carried out using Intercooled Stata version 11 (College Station, TX) or SAS version 9 (Cary, NC). Power calculations are presented in Section C1.

***5. Informed Consent Document*** – Since there is no direct contact with study participants, no informed consent documents will be used for this study.

***6. Risks/Benefits*** – There will be no direct human subject contact in this proposed research project. This project is a secondary data analysis, in other words, no primary data will be collected. All data will be obtained from the Texas DSHS and publically available data sources (e.g., HAPs). All information will be kept confidential according to HIPAA policies and procedures. Therefore, the potential risk involved will be minimal. The major risk is loss of confidentiality. This risk will be minimized by reporting results only in the aggregate.

All data will be stored on password and firewall protected Network-Attached Storage servers on The University of Texas Health Science Center at Houston network. Further, the data will be destroyed upon completion of project. Specifically, it is anticipated that the project will be completed by summer 2013. At that time, the data will be deleted from all computers and any compact discs will be shredded. The knowledge gained from this study will improve our understanding of the determinants of infant biochemical profiles and potential risk factors for birth defects. Little is known regarding the causes of birth defects, therefore future prevention efforts rest on the knowledge gained from studies seeking to elucidate the etiology of these conditions.

***7. Student Investigator*** – Not applicable.

***8. Other Considerations*** – The principal investigator and all collaborators have extensive experience with registry data, including GIS information.
